# Regulatory Effects of Cu, Zn, and Ca on Fe Absorption: The Intricate Play between Nutrient Transporters

**DOI:** 10.3390/nu5030957

**Published:** 2013-03-20

**Authors:** Nathalie Scheers

**Affiliations:** Division of Life Sciences, Food Science, Department of Chemical and Biological Engineering, Chalmers University of Technology, SE-412 96 Gothenburg, Sweden; E-Mail: nathalie.scheers@chalmers.se; Tel.: +46-31-772-3821; Fax: +46-31-772-3830

**Keywords:** iron, absorption, Fe, Zn, Cu, Ca, ascorbate

## Abstract

Iron is an essential nutrient for almost every living organism because it is required in a number of biological processes that serve to maintain life. In humans, recycling of senescent erythrocytes provides most of the daily requirement of iron. In addition, we need to absorb another 1–2 mg Fe from the diet each day to compensate for losses due to epithelial sloughing, perspiration, and bleeding. Iron absorption in the intestine is mainly regulated on the enterocyte level by effectors in the diet and systemic regulators accessing the enterocyte through the basal lamina. Recently, a complex meshwork of interactions between several trace metals and regulatory proteins was revealed. This review focuses on advances in our understanding of Cu, Zn, and Ca in the regulation of iron absorption. Ascorbate as an important player is also considered.

## 1. Introduction

Iron (Fe) is the second most abundant metal on earth and is a necessity for all life. Iron plays the key role in numerous enzymatic reactions due to its ease in shifting between the two common oxidation states, ferrous (Fe^2+^) and ferric (Fe^3+^) iron. In humans, iron is the carrier of oxygen by means of hemoglobin in erythrocytes and myoglobin in myocytes, and is therefore indispensable for providing oxygen to the tissues. Severe iron deficiency leads to anemia with a number of symptoms such as tiredness, palpitations, and other signs associated with impaired oxygen transport. Also, iron overload is linked to several diseases involving functional changes of hemoglobin, such as thalassemia and sickle cell disease. Iron in excess is detrimental because of its propensity to cause oxidative damage. Except for the sloughing of epithelial cells, which removes iron confined in the absorptive cells, there is no other way to eliminate iron. It is therefore of major importance to tightly regulate iron absorption.

## 2. Dietary Iron and Bioavailability

There are mainly two types of iron in food, heme iron and non-heme iron. Heme iron is highly abundant in meat (myoglobin) and liver (hemoglobin). Heme iron is coordinated to a porphyrin ring deeply hidden inside a globular protein, which makes the access to iron restricted to small molecules such as O_2_ and CO. Fortunately, the arrangement also protects iron from forming insoluble precipitates in the intestine and thus promotes iron bioavailability. Non-heme iron is present in vegetables, cereals, and fruits, which constitute the major part of a healthy diet. In the human intestine, non-heme iron is mostly present in soluble or insoluble chelate complexes. Organic acids such as ascorbic acid or citric acid form chelates with iron and enhance iron absorption [1]. The diet also contains inhibitors of iron absorption. Phytate is abundant in legumes, cereals, and nuts and is considered to be the most powerful anti-nutrient due to its high binding capacity for metals and also its ability to form large insoluble aggregates [2]. Since non-heme iron is absorbed as single ions, there are several parameters that determine the extent of absorption. In addition to the interactions with organic compounds, non-heme iron transport is pH-dependent and occurs in competition with other divalent ions to be transported by metal transmembrane carriers. To complicate things further, intracellular effects and factors specific to the host determine iron absorption. 

### Iron Requirements

An average individual contains about 50 mg Fe/kg, which is approximately 3.5 g for a 70 kg-person [3]. About 65% of these 3.5 g exist as hemoglobin-iron in erythrocytes. The remaining iron is present in myoglobin of muscle fibers, enzymes in various tissues, and stored in macrophages, liver, and bone marrow. After cellular turnover, most of the body iron is recycled primarily by macrophages of the reticuloendothelial system. Cellular sloughing into the gastrointestinal tract, bleeding, and respiration cause losses of 1–2 mg Fe per day. The iron levels are restored by intestinal absorption to maintain the balance. A typical western world diet provides 10–18 mg of iron per day. About 10% is absorbed, which does not give much of a margin in times of increased iron requirements, as in pregnancy or fast-growing children. 

## 3. Iron Transport in and across the Human Enterocyte

### 3.1. Iron Influx

Heme-bound iron may be absorbed from the gastrointestinal lumen through the heme-carrier protein HCP1. HCP1 is primarily a H^+^-coupled folate transporter suggesting that absorption of heme-iron is affected by folate availability [4]. The absorbed heme-bound iron must be released in the cytosol by means of heme oxygenase [5] in order to join the same pathway as elemental iron. In addition, heme is likely to be absorbed via receptor-mediated endocytosis, which was suggested already in 1979 [6], but the mechanism still remains elusive. 

DMT1 (NRAMP2/SLC11A2/DCT1), the main transporter for divalent cations, transports elemental iron across the lumenal membrane of absorptive cells. Human DMT1 (hDMT1) simultaneously imports hydrogen (H^+^) and ferrous iron (Fe^2+^) [7]. It is still debated whether hDMT1 is specific for Fe^2+^ or also transports other metal ions [8]. Although, there is convincing evidence that hDMT1 transports Fe^2+^ in preference to Zn [7,9,10,11]. However, Cd^2+^ is preferred to Fe^2+^ [11]. hDMT1 is expressed in four different isoforms (DMT1A, DMT1A-IRE, DMT1B and DMT1B-IRE), which are differently regulated and distributed [12]. DMT1A and DMT1A-IRE are mainly concerned with the apical influx at polarized enterocytes while the DMT1B isoforms are involved in cytosolic iron sequestration from endosomal import by transferrin receptors at the basal membrane. Studies in the mouse have indicated that DMT1 is expressed along the entire small intestine but is only increased in the proximal part of the duodenum in response to iron starvation [13] suggesting that the proximal duodenum is the major site for iron absorption. Since elemental iron is mostly transported into the enterocyte through DMT1, the transport efficiency will be partly determined by the availability of the ferrous form. The availability of Fe^2+^ is assured by the expression of a brush border-associated ferric reductase. 

### 3.2. Influx Promotion

Duodenal cytochrome b (DCYTB/CYBRD1) has been proposed as responsible for the reduction of ferric iron in humans [14]. DCYTB is a heme-containing trans-membrane protein that reduces lumenal Fe^3+^ to Fe^2+^ by means of an electron donated from ascorbate in the cytosolic compartment. It has been observed that DCYTB co-localizes with DMT1 at the lumenal membrane, and the levels are increased in iron-starved cells in the rat [15] and in humans with iron deficiency anemia [16]. It has been shown that reductase activity is required for elemental iron uptake in human Caco-2 cells [17]. In contrast, the presence of DCYTB in the duodenum of the mouse does not seem necessary for iron absorption [18]. In this context, it should be emphasized that mice endogenously produce ascorbate, which may aid in reducing iron for the uptake by DMT1. More than a decade ago, it was suggested that iron as Fe^3+^ could be transported across the enterocyte membrane by means of an integrin-mobilferrin-paraferritin (IMP) complex in the rat [19]. This work has not been reproduced so far. If it exists, the IMP-pathway may be the answer to why reduction of ferric iron by DCYTB is redundant in the mouse. The relevance of the IMP-pathway to humans has not yet been established. 

### 3.3. Intracellular Transport and Efflux

In the cytosol, newly absorbed iron initially joins the labile iron pool (LIP) also referred to as the chelatable iron pool, which consists of metabolically active forms of iron mainly loosely associated with macromolecules. Depending on the cellular requirements, iron may be distributed from the LIP to the major intracellular iron storage protein, ferritin, where iron is stored inside the hollow core as ferric phosphate salts. The ferritin molecule consists of L and H subunits, in which the H subunits possess ferroxidase activity required for the acquisition of iron salts. It has been proposed that a recently identified cytosolic iron chaperone, PCBP1 (hRNP/α-CP1), is responsible for the incorporation of iron to ferritin [20]. However, it is not yet fully clear how the iron delivery to different intracellular compartments occurs. In times of systemic shortage, iron is transported directly to the basal membrane for transmembrane export to the vascular compartment by the ferrous iron exporter ferroportin (SLC40A1/FPN1/IREG1) [21]. Iron is transported across the membrane as Fe^2+^ and is then oxidized to Fe^3+^ by a membrane-bound ferroxidase, hephaestin. The ferric iron is transferred to transferrin (Tf) for systemic transportation.

## 4. Systemic Regulation of Iron Absorption

### 4.1. Control of Iron Release

**Figure 1:**
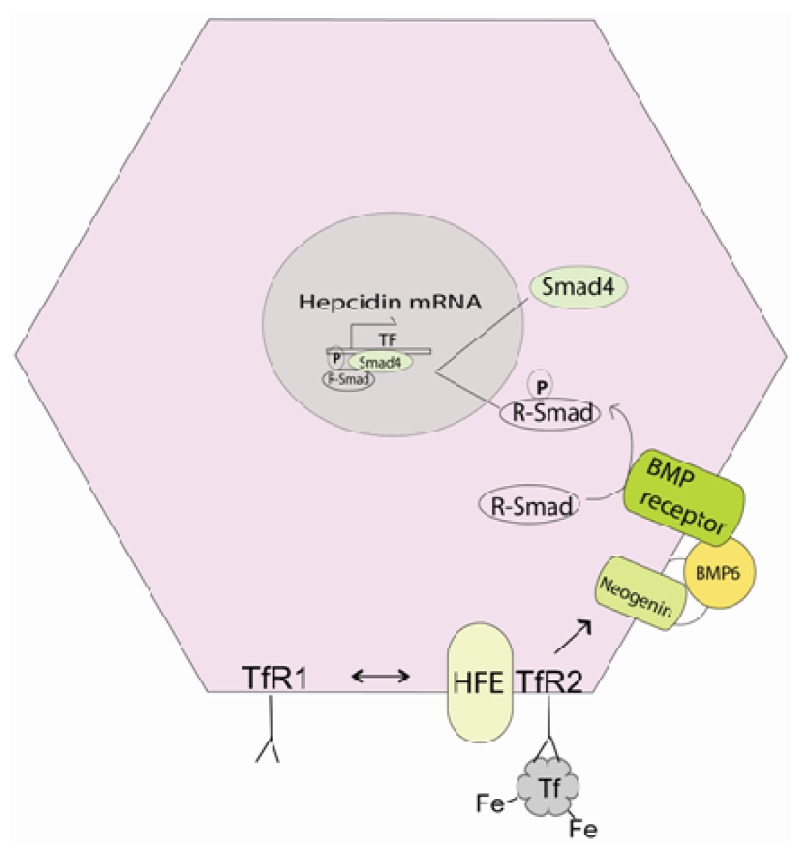
Overview of the SMAD/BMP pathway in hepatocytes. Iron-saturated transferrin acts like a hepcidin transcription switch. Low levels of iron-saturated transferrin destabilizes the TfR-HFE complex, preventing the phosphorylation of R-SMAD, which represses hepcidin transcription.

Systemic iron homeostasis must be rigorously controlled, not only because iron is essential for survival, but also since iron is highly toxic if overloaded. Hepcidin, a small peptide hormone produced in the liver, is the key regulator of systemic iron levels. Hepcidin controls iron release from the enterocytes into the systemic circulation by binding to the iron exporter ferroportin, thereby subjecting ferroportin to ubiquitin-dependent degradation [22]. Recently, it was shown that hepcidin-mediated ferroportin degradation does not require the induction of the JAK/STAT pathway [23] as previously described. The removal of ferroportin at the enterocyte basal lamina blocks iron efflux and as a consequence enterocyte levels of ferritin increase, which reflect the intestinal iron load. Hepcidin is feedback-regulated by iron itself and is decreased during iron deficiency and increased in iron overload. The effect is thought to be mediated by the BMP pathway [24,25]. Circulating iron-saturated transferrin interacts with transferrin receptor 2 (Tfr2) at the surface of hepatocytes, which induces hepcidin production through interaction of BMP6 and SMAD signaling (Figure 1). When iron-saturated transferrin levels are low the required stabilization of Tfr2 by the associated membrane protein HFE is lost and thus transcription of hepcidin mRNA is repressed. Recent data suggest that Tfr2 and HFE associate with another protein, hemojuvelin (HJV), to form a membrane-interacting complex [26]. At present, there are no indications of the importance of this complex for the regulation of hepcidin transcription. Hypoxia also regulates hepcidin expression in hepatocytes via the SMAD/BMP pathway [27]. The hypoxia-inducible factor 2 (HIF2) has been proposed as a mediator, however HIF2 was recently observed as not being involved in the regulation of hepcidin transcription [28,29].

Systemic iron is also absorbed in hepatocytes as nontransferrin-bound iron (NTBI), which requires the usual route of reduction to ferrous iron by membrane-bound reductases and uptake through metal transporters. DMT1 is generally expressed in all tissues and may therefore play a role in hepatocyte iron influx. The zinc transporter ZIP14 is abundantly expressed in liver cells and has been shown to be involved in NTBI uptake [30]. Later studies in the *Xenopus laevis* oocyte expression system have revealed that ZIP14 imports Fe^2+^ in addition to Zn, Cd and Mn. Interestingly, the transport of Fe^2+^ was dependent on Ca^2+^, but not on Zn^2+^ [31]. The authors suggest that ZIP14 may have two connected metal translocation pathways, which may explain the former. It has also been observed that ZIP14 protein expression is down-regulated by the abundance of the HFE protein in liver cells, inhibiting the uptake of iron [32]. When ZIP14 expression was silenced there was no effect of HFE levels on NTBI uptake.

In summary, there are several indications of the involvement of ZIP14 in the regulation of hepcidin control of iron release. There are other zinc transporters currently under investigation, e.g., several zinc importers (rZIP5, rZIP6, rZIP7, and rZIP10) in rat liver cells have been shown to be affected by iron status, but their role in regulating iron absorption is unclear [33].

### 4.2. Iron Absorption and Inflammation

Hepcidin is an important mediator in the acute phase reaction of inflammation [34]. Several conditions including inflammatory diseases and systemic infections are associated with hypoferremia and increased hepcidin levels. It may be beneficial to limit the iron supply to prevent further reproduction of the infecting microorganism during infection or to decrease iron-mediated oxidative damage of inflamed tissues. In these conditions, increased hepcidin levels are caused by activation of the JAK/STAT pathway mediated by the inflammatory cytokine IL-6 [35]. Hepcidin-independent regulation of ferroportin in patients with the ferroportin mutation D157G has been reported [36]. It was suggested that the D157G mutated ferroportin is phosphorylated by JAK2, which would induce the degradation of ferroportin independent of ubiquitin. In summary, it seems likely that normal regulation of systemic influx of dietary iron by hepcidin is mediated by the BMP pathway while the onset of JAK/STAT signaling is induced in times of extraordinary stress in which the effects of the BMP pathway need to be overridden.

### 4.3. Recycling of Iron by Macrophages

Macrophages play an important role in executing the regulatory events leading to changes in systemic iron levels. Senescent or damaged erythrocytes are removed from the circulation by phagocytosis. Heme-iron is transported from the phagocytic vesicles into the cytosol by means of a transmembrane permease, HRG1 [37]. Elemental iron is released by means of DMT1 into the cytosol where it associates with the LIP or is incorporated into ferritin. Macrophages also scavenge iron by receptor-mediated endocytosis of haptoglobin-hemoglobin complexes or hemopexin-heme complexes retrieved from ruptured erythrocytes. Iron is eventually exported through ferroportin, which is partly controlled by hepcidin. In addition, the porphyrin ring of heme regulates the transcription of ferroportin by activating Nuclear Factor Erythroid 2 (NRf2) control of the ferroportin promoter [38]. This further strengthens the crucial role of hepcidin/ferroportin in regulating systemic iron levels.

## 5. Regulation of Iron Transport at the Enterocyte Level

### 5.1. Iron Regulatory Protein 2 (IRP2) Senses Cellular Iron Status

The expression of iron transporters is regulated on the mRNA level by means of common motifs, iron responsive elements (IREs) [39]. Ferritin and one of the isoforms of ferroportin mRNA both contain an IRE sequence within the 5′ untranslated region (5′ UTR). DMT1A-IRE and DMT1B-IRE possess an IRE in the 3′ UTR. When cellular iron levels are low, Iron regulatory proteins (IRPs) bind to IRE sequences in the 5′ UTR of the ferritin and ferroportin mRNAs, which block the translation. Binding to the 3′ IRE on DMT1 mRNA stabilizes the transcript, which promotes protein translation and increases the lumenal absorption of iron. In times of adequate iron absorption, the elevated levels of cytosolic Fe in the LIP stimulate the proteasomal degradation of IRP2 [40,41], which increases ferroportin levels and the cellular efflux of iron to the systemic circulation.

There are two forms of IRPs; IRP1 and IRP2. Both IRPs are RNA-binding proteins. IRP1 also function as a cytosolic aconitase and it appears that this is its normal state in animal tissues. The mRNA binding of IRP1 does not increase in iron-deficient mice, despite the activation of IRP2 [42]. In our own studies in intestinal Caco-2 cells we observed increased IRP2, but not IRP1 levels in iron-deficient cells, supporting the former statement [43]. Also, IRP2 binding activity is increased when IRP1 activity is lost, as in IRP1^−/−^ mice, thus compensating for its absence [42]. The IRPs are differently expressed throughout the body. IRP1 is mainly present in tissues such as brown fat and kidney in the mouse, in which IRP2 is expressed in all tissues. 

Two of four isoforms of DMT1 [12] and one of two forms of ferroportin [44] do not contain the IRE sequence in their mRNA transcripts and thus are not under the control of IRPs. This could potentially mean that the regulation of iron absorption by IRP2 may be overridden when the circumstances require it. In fact, ferroportin 1B (FPN1B) lacks the IRE motif and is not repressed in iron deficiency [44]. 

### 5.2. The Importance of Cu for Iron Release

It is well known that individuals exposed to a copper-deficient diet develop iron deficiency anemia as well as iron overload in specialized tissues such as the liver and intestine. The iron and copper homeostases are linked by the inability to export Fe in the absence of Cu to the systemic circulation. However, Cu deficiency does not affect ferroportin expression [45]. Ferroportin activity is tightly controlled by the ceruloplasmin-homologue hephaestin [46]. Hephaestin is an integral transmembrane ferroxidase, which co-migrates with ferroportin to the basal membrane, in response to increased intracellular iron levels, in which they form a complex [47,48]. Exported ferrous iron requires oxidation to ferric iron, which is accomplished by the Cu-dependent ferroxidase activity of hephaestin. Ceruloplasmin is another Cu-dependent ferroxidase important for iron metabolism. This is evident in the rare disease aceruloplasminemia, in which the absence of serum ceruloplasmin results in decreased mobilization of iron from body stores giving symptoms related to anemia. It was recently shown that serum ceruloplasmin levels increase during iron deficiency in mice [49]. Also, increased Cu consumption by the iron deficient mice raised the serum ceruloplasmin activity suggesting a compensatory route for increasing systemic iron levels in times of deprivation.

**Figure 2:**
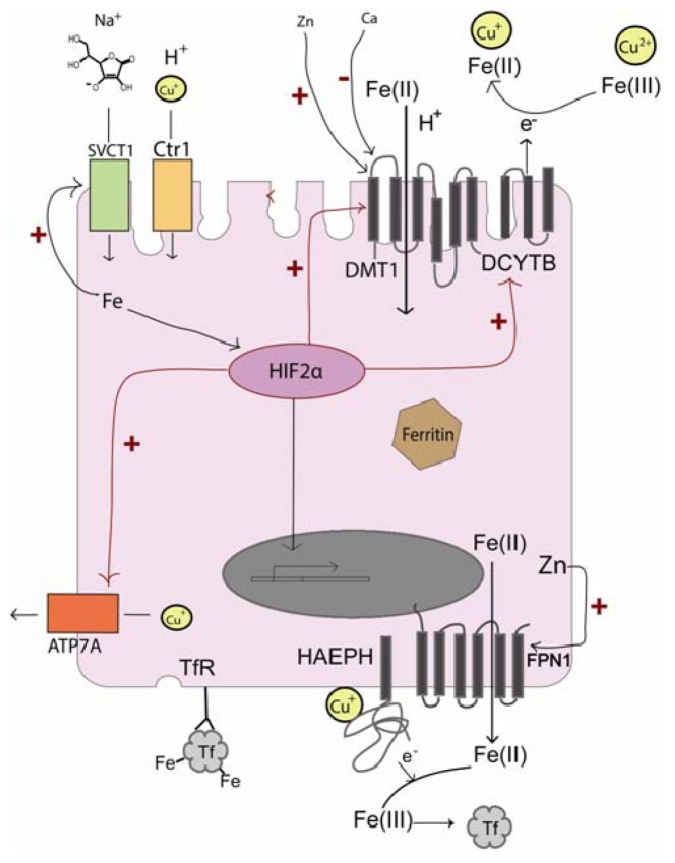
Overview of interactions between Fe, Cu, and ascorbate transporters with the hypoxia transcription factor HIF2α, Fe, Cu, Zn, and Ca in enterocytes. Cu transport and the control of transcription by HIF2α is central for regulation of iron absorption.

It was previously thought that the iron transporter DMT1 could be involved in the transport of Cu^+^ across the apical border of the intestinal epithelium. Recent data indicate that DMT1 does not transport Cu^+^ [11]. It has been established that Cu is absorbed in the intestinal lumen by the human copper transporter hCtr1 [50]. hCtr1 transports Cu in its reduced form, Cu^+^, and has been proposed to require the assistance of the ferrireductase Dcyb, which also functions as a cupric reductase [51]. In this manner, Cu^+^ and Fe^2+^ availability is linked on the lumenal level in addition to the systemic level, further strengthening the co-regulation of these two micronutrients (Figure 2). 

### 5.3. Transcriptional Regulation of Fe and Cu Transport

Recent findings have shown that DMT1 and DCYTB are regulated on the transcriptional level by the hypoxia-induced transcription factor HIF2α [52,53]. Both DMT1A and DCYTB were found to have HIF responsive elements (HREs) in their respective regulatory genes and HIF2α-mediated transcription was induced by a low-iron diet in mice [52]. HIF2α also induces ferroportin expression in iron deficient mice [54]. HIF2α then regulates both Fe import and export, indicating its importance for fast track regulation of systemic iron levels. 

In summary, during low enterocyte iron levels, HIF2α up-regulates iron import from the intestinal lumen, at the same time preparing for the incoming iron to be exported across the basal membrane, with the acquisition of copper by up-regulating the human copper transporter 1 (hCtr1) [55]. The increased intracellular copper level increases the expression/stability of hephaestin, which facilitates the required cooperation with ferroportin in iron export into the systemic circulation. HIF2α and Cu modulates the mRNA levels of the copper exporter Atp7a (Menkes copper ATPase) possibly as a negative feedback mechanism in response to high copper levels [56,57]. 

### 5.4. The Emerging Role of Ascorbate

Ascorbate is an important regulator of iron transport; it assists DCYTB in the intestinal lumen by reducing ferric iron to the bioavailable ferrous form. In addition, cellular ascorbate status influences enterocyte protein levels of DMT1 and DCYTB in the absence of iron [58]. HIF2α, which regulates DMT1, DCYTB, and ferroportin transcription, is degraded in response to ascorbate, iron and oxygen. Also, low ascorbate, iron and oxygen levels stabilize HIF2α, providing a possible mechanism for the effects of ascorbate on DMT1 and DCYTB expression. Ascorbate may be important for the ferrireductase activity of DCYTB, as it has been suggested to donate electrons to the cytoplasmic domain for transmembrane propagation, taking part in the lumenal reduction of iron [59]. DCYTB resembles Cytochrome b561 for which an ascorbate-dependent electron shuttle has been described [60]. It has been suggested that DCYTB also reduces copper in addition to iron, further indicating the regulatory role of copper in iron transport [51]. Ascorbate is absorbed in the intestinal lumen by means of the ascorbate/sodium transporter SVCT1. The SVCT1 expression is regulated by ascorbate [61] and also by iron [62]. Increased cellular iron levels up-regulate ascorbate uptake. Increased ascorbate levels then down-regulate DCYTB and DMT1 in response to the iron load, closing the feedback loop. 

### 5.5. Zn Transport

Iron down-regulates its own uptake by the DMT1 transporter while zinc up-regulates uptake of iron by increasing the protein levels of DMT1 in human Caco-2 cells [9]. The basolateral efflux of iron and the mRNA expression of ferroportin are also increased by zinc supplementation. Zinc promotes ferroportin transcription by stimulating the binding of Metal transcription factor 1 (MTF-1) to the ferroportin promoter [63]. As described earlier, iron is imported to hepatocytes via ZIP14 [30], which is also expressed in intestinal cells. In the state of inflammation when iron absorption is inhibited and iron is removed from the circulation, circulating zinc is removed to prevent its stimulation of iron absorption and efflux by ferroportin. The ZIP14 transporter in hepatocytes accomplishes the systemic removal of zinc and it is up-regulated by the acute phase cytokine interleukin-6 [64], further stating the central role of interleukin-6 in promoting anemia of inflammation.

The Zip4 transporter is expressed along the intestine and is crucial for zinc absorption and a normal intestinal epithelium [65]. However, a specific role of the Zip4 transporter in iron absorption has not been established.

### 5.6. Ca in Iron Absorption

Calcium is generally believed to interfere with iron absorption. It has also been established that this effect requires high Ca intake and occurs at the lumenal level. The mechanism involves the iron transporter hDMT1, of which transport is inhibited by Ca^2+^ [10]. Ca^2+^ inhibits Fe^2+^ transport non-competitively with low affinity. It is not clear how Ca^2+^ interferes with iron transport, but voltage dependence or intracellular Ca^2+^ signaling seems to be ruled out [10]. Iron uptake, as indirectly estimated by intracellular ferritin content in Caco-2 cells, is only decreased with high concentrations (1.25–2.5 mM) of Ca^2+^ after a long exposure time (16–24 h) [66]. The membrane expression of hDMT1 decreased accordingly, suggesting that the inhibition of iron uptake may involve regulation of transporter abundance. It has also been reported that short time Ca^2+^ incubations (1.5 h) of human intestinal Caco-2 cells down-regulated ferroportin levels at the basal membrane and the associated iron efflux [67]. The ferroportin levels were restored after 4 h, suggesting that the calcium effect is of short duration.

Several studies in humans have shown a correlation with Ca and decreased Fe absorption. However, long-term high Calcium intake does not correlate with impaired iron status, as concluded by Lönnerdal (2010) in an extensive review on the subject [67].

## 6. Conclusions

In recent years, substantial progress in the understanding of iron absorption has been made. It is clear that the absorption of iron is extremely complex and it is becoming increasingly difficult to separate the absorption by several players into individual events; when can we actually state that an iron deficiency is primary? We are still lacking pieces of information, which may be the explanation for discrepancies encountered. New studies are frequently published in the field, giving hope that gaps eventually will be filled. However, plenty of additional work is required. 

It is clear that Cu^+^ is central to iron absorption and that there is no competition with Fe^2+^ for DMT1 transport. Also, the reduction of both ions by DCYTB shows that Cu^+^ and Fe^2+^ uptake into the enterocyte is co-regulated. In addition, Cu is required for the efflux of Fe^2+^ through ferroportin, further indicating that their absorption is mutually inclusive. 

Zinc deficiency has been extensively investigated particularly due to its immune modulatory function and its appearance in other micronutrient deficiencies. Zinc stimulates iron absorption but does not seem to be a prerequisite to maintain normal iron levels. Cow’s milk was, or still is, widely used as a food supplement for small children and is associated with low iron absorption. This may not be an effect of the calcium content. On the mechanistic level, neither zinc nor calcium seem to be as crucial for iron absorption as copper, whilst copper deficiency and sufficient copper levels in the diet have been the least investigated. The same refers also to ascorbate and its intracellular effects on iron absorption. 
